# Physicochemical evaluation and Fourier transform infrared spectroscopy characterization of quality protein maize starch subjected to different modifications

**DOI:** 10.1111/1750-3841.15391

**Published:** 2020-08-27

**Authors:** Olugbenga Olufemi Awolu, Joshua Wisdom Odoro, Jumoke Bukola Adeloye, Oluranti Mopelola Lawal

**Affiliations:** ^1^ Department of Food Science and Technology Federal University of Technology Akure Nigeria; ^2^ Food Quality and Design Group Wageningen University and Research Gelderland The Netherlands

**Keywords:** Starch, FTIR, Quality Protein Maize, Modification, pasting properties

## Abstract

**Abstract:**

Quality protein maize (QPM) is a biofortified maize rich in lysine and tryptophan, essential amino acids required in human nutrition. This research therefore characterizes native and modified starches from QPM by evaluating the physicochemical properties, Fourier transform infrared spectra (FTIR), and pasting properties. The native QPM starch was modified by oxidation, acetylation, pregelatinization, and acid thinning techniques. The starch yield of native QPM was 43.80%, while that of modified starches were from 88.22 to 98.34%. The moisture content of the native and modified starches was from 4.56 to 9.20 g/100g. Modifications significantly (*P* ≤ 0.05) reduced the lipid, protein, and amylose contents of the QPM. While the native starch had 0.72 g/cm^3^ bulk density, modified starches were between 0.59 and 0.88 g/cm^3^; chemical modification reduced the bulk density and physical modification increased it. In addition, all the modifications except oxidation significantly (*P* ≤ 0.05) increased water absorption capacity. The oil absorption of the starch samples was increased by modification techniques used with the exception of physical modification. Chemical modification reduced the viscosity of QPM starch while physical modification increased it. The reducing sugar content of the starch was reduced by both the physical and chemical starch modification techniques. Acetylated sample exhibited the highest swelling power while acid‐thinned sample had the least. The major functional groups identified via FTIR were OH, C‐H, C=H, and C≡H. Modifications did not affect the functional groups as all the native and modified starches (except oxidized sample) all have similar spectrum and bands stretch.

**Practical Application:**

The study contributes to existing knowledge on valorization of modified starch from quality protein maize. Profiling the chemical attributes of modified starches is especially valuable in novel food processing techniques.

## 1 INTRODUCTION

Humans and animals derive their energy from starch and it is a predominant food reserve substance found in green plants. It also serves as an energy reservoir which is readily converted into useful chemicals products, hence, it has found numerous application in many industries (Abbas, Khalil, & Hussin, 2010). Food products with abundant starch include maize, rice, wheat, cassava, yam, and potato, in addition to other sources which includes millet, oat, sorghum, lentils, and peas. Until recently, starches have been explored from seeds of fruits and their starches have been explored by several researches (Cisse, Zoue, Soro, Megnanou, & Niamke, 2013; Hassan et al., 2013; Olatunde, Arogundade, & Orija, 2017).

The matured maize kernel comprises about 70% starch of the dry weight making the maize starch of major economically important component of the crop (Ji et al., 2003). Native starch is made up of approximately 75% branched amylopectin and 25% amylose which is either linear or slightly branched. Amylose is a linear polymer and has its makeup from glucopyranose units linked through α‐d‐(1,4) glycosidic linkages, while the amylopectin fraction of the starch is of a highly branched polymer which has one of the highest molecular weights recognized among naturally occurring polymers (Karim, Norziah, & Seow, 2000).

Researches have proven that native starches have distinctive properties, but they lack flexibility in the food and nonfood industries of today where these native starches are expected to have freeze‐thaw strength, improved swelling capacity, better gelling properties, temperature stability, enhanced viscosity, increased or decreased digestibility, and also be able to imitate the properties of gelatin and certain fats in food production or formulation (Abbas et al., 2010). In the light of this, it is believed that starches from quality protein maize (QPM) might possess an increased amylopectin content, higher swelling capacity, and a lower risk of getting retrograded (Cisse et al., 2013). Interestingly starch can be chemically, physically, or enzymatically modified. Physical modification is achieved by using moisture and heat (pregelatinization) to treat the starch while chemical methods involve esterification, etherification, and crosslinking or decomposing the starch by acid or enzymatically hydrolyzing it which will result in starch molecules possessing additional functional groups and distinctive features (Singh, Kaur, & McCarthy, 2007).

Several studies have been conducted on legume starches, including native and modified forms (Adebowale, Afolabi, & Lawal, 2002). Some legumes extensively studied were Bambara groundnut (Adebowale et al., 2002), great northern bean (Sathe & Salunkhe, 1981). QPM is a biofortified food and its nutritional profile has been improved using conventional breeding techniques (Cisse et al., 2013). QPM has lower protein content of only about 9% compared to legumes (around 45%), and carbohydrate content of about 74%. The high starch content of QPM is an indication that its native and modified forms are worth being studied. In addition, QPM is an entirely different species from other maize species that their starches have been studied.

The study was meant to investigate the impact of some physical and chemical modification techniques on physicochemical and functional properties, as well as the FTIR of QPM native and modified starches in order to assess the starch qualities.

## 2 MATERIALS AND METHODS

### 2.1 Materials

QPM was purchased from the Institute of Agricultural research and Training Centre (IAR&T), Ibadan Oyo State, Nigeria. All chemicals and reagents used were of analytical grade.

### 2.2 Experimental design

All starch samples were prepared according to AOAC (2005) method and experiments were carried out in triplicates with the exception of pasting characteristics and Fourier transform infrared spectra.

### 2.3 Starch isolation

Maize kernel of about 1 kg was properly rinsed and steeped in distilled water containing 0.1% sodium metabisulphite overnight in a refrigerator. The kernels were blended at low speed, 100 rpm for 5 min. The resultant slurry was sieved through 100 mesh until the waste solution was cleaned and the mixture was evenly dispersed in 4% NaCl solution. The starch suspension was left overnight in a refrigerator (4 °C), washed and centrifuged (0502‐1, Hospibrand, USA) at 3,000 × *g* for 20 min. The white sediment was dried at 40 °C in a convection oven (Uniscope, SM9053, England) for 24 hr, ground with an attrition mill, and hurled past a 100‐mesh screen and stored in sealed polyethylene bag at room temperature (25 to 30 °C) prior to further analyses (Waliszewski, Aparicio, Bello, & Monroy, 2003).

### 2.4 Acetylation of native starch

The method of Lawal and Adebowale (2005) with slight modifications was used. About 100 g of starch was dispersed in 500 mL of distilled water; it was stirred magnetically for 20 min. The pH of the slurry was adjusted to 8.0 using 1 M NaOH. About 10.2 g acetic anhydride was added over a period of 60 min, while maintaining a pH range of 8.0 to 8.5. The reaction was allowed to proceed for 5 min after the ample addition of the whole acetic anhydride. The pH of the slurry was adjusted to 4.5 using 0.5 M HCl, filtered, washed four times with distilled water, and air‐dried at 30 ± 2 °C for 48 hr, and packed in a polyethylene bag prior to further analyses.

### 2.5 Oxidation of native starch

The method adopted by Awolu and Olofinlae (2016) was employed. About 100 g of starch was dispersed in 500 mL of distilled water. The pH was adjusted to 9.5 with 2 M NaOH. Sodium hypochlorite (NaOCl) (10 g) was added to the slurry over a period of 30 min, while maintaining a pH range of 9.0 to 9.5, with constant stirring using a magnetic stirrer at 30 °C. The reaction proceeded for 10 min after the final addition of NaOCl while the pH was later adjusted to 7 with 1 M sulfuric acid. The oxidized starch was filtered, washed four times with distilled water, and air‐dried at 30 °C for 48 hr. It was packaged in a polyethylene bag for further analyses.

### 2.6 Acid thinning of native starch

The method of Lawal and Adebowale (2005) was employed with slight modifications using 0.15 M concentration of Hydrochloric acid (HCl). About 100 g of the native starch was made into a slurry by dissolving it in 500 mL of 0.15 M HCl. The mixture was stirred with a magnetic stirrer for 8 hr, while maintaining a temperature of 50 °C. The acid modified starch was filtered and the residue obtained was washed four times with distilled water. It was dried in the hot air oven for 48 hr at 30 ± 2 °C. The starch was packaged in a polyethylene bag for further analyses.

### 2.7 Pregelatinization of the native starch

The method of Yousif, Gadalla, and Sorour (2012) was employed for pregelatinization. Starch solution of ratio 1:1 (300 g starch + 300 mL deionized water) was incubated at 63 °C for 5 min. The gelatinized starch was produced by drying the solution in the oven at 30 °C for 24 hr. It was packaged in a polyethylene bag for further analyses.

### 2.8 Starch yield determination

The starch yield was evaluated as described by Awolu and Olofinlae (2016). It is the percentage ratio of starch recovered after extraction to the sample. The starch yield was estimated using the equation,

$$\mathrm{Starch}\;\mathrm{yield}\;=\frac{\mathrm{weight}\;\mathrm{of}\;\mathrm{starch}\;\left(g\right)}{\mathrm{weight}\;\mathrm{of}\;\mathrm{sample}}\;\;\times \;100$$

### 2.9 Proximate composition of starch samples

The proximate composition, including moisture, ash content, crude protein, crude fat, and crude fiber of the starches, were determined using AOAC (2005) methods. The carbohydrate was estimated by difference.

### 2.10 Determination of acetyl group, carboxyl, and carbonyl contents

The methods of Lawal and Adebowale (2005) were employed for the determination of acetyl group, carboxyl, and carbonyl contents of the native and modified starches.

### 2.11 Determination of amylose and amylopectin contents

The method of Williams, Wu, Tsai, and Bates (1958) was used for the determination of the amylose and amylopectin contents of the native and modified samples.

### 2.12 Determination of pH

The pH meter was calibrated with KOH buffer solutions of pH 7.0 and 4.0 before the measurements. The modified and native starch samples (5 g) were weighed in triplicate into a beaker, mixed with 20 mL of distilled water. The resulting suspension was stirred for 5 min and left to settle for 10 min. The pH of the water segment was determined using the calibrated pH meter (pH–107 Sinotester) (Ashogbon & Akintayo, 2014).

### 2.13 Determination of the functional properties of the native and modified samples

The *bulk densities* of the modified and native starches were determined by the method of Ashogbon and Akintayo (2014). *Water and oil absorption capacities* were determined by the procedure used by Yousif et al. (2012). *Least gelation concentration* was determined by the method of Sathe and Salunkhe (1981). *Dispersibility* was determined by the method described by Akanbi, Nazamid, and Adebowale (2009). *Sedimentation* was determined by the method of Rafiq, Jan, Singh, and Saxena (2015).

### 2.14 Effect of temperature and pH on swelling power and starch solubility

The effects of temperature and pH on the swelling power and starch solubility of the starch samples were determined using Sathe and Salunkhe (1981) method.

### 2.15 Starch pasting properties

Pasting properties of the starch samples were evaluated using 80 g/L dispersion of the starch in distilled water. A Brabender viscograph (Type VA–V, Brabender GmbH, Duisburg) equipped with a 700 µg sensitivity cartridge was used for this study. The temperature of the slurry was raised from 30 to 95 °C, and kept at this temperature for 30 min, before cooling down to 50 °C. A constant rotational velocity of 75 rpm was maintained and the heating or cooling rate was 1.5 °C/min throughout the process.

### 2.16 Starch viscosity measurement

The starch suspension of 5% w/v was heated to 90 °C for 30 min in a temperature controlled digital electric water bath (Model DK–420) with continuous stirring. The paste was transferred to a rotatory viscometer (Viscotester VT–04E Rion co, Ltd, Tokyo, Japan) using the rotor No. 1. Paste viscosity was measured from 90 to 30 °C cooling paste.

### 2.17 Fourier transform infrared spectroscopy of starches

The native and modified QPM starches were dried to constant weight and the samples were labelled and measured to equal weights. The starch samples were then treated with equal quantity of potassium bromide (KBr) salt, then each sample was pressed in KBr Salt Plates and transferred to the Fourier transform infrared spectrometer (FTIR Spectrometer, Brucker, Germany). The instrument was on and kept to optimized and self‐set for about 15 min before usage. The scan button was pressed to start the process and various spectra for each functional group was displayed on the screen and a recording device notes the results.

### 2.18 Color of starch samples

The color of the starches was measured using a Color Meter PCE–CSM 2 (Deutschland GmbH) connected to a CQCS3 software. The spectrophotometer was calibrated against a white plate before the reading was taken. The parameters recorded were *L* ^*^, *a* ^*^, and *b* ^*^ coordinates of the CIE scale.

### 2.19 Statistical analysis

The results obtained was analyzed statistically using SPSS (v.21, IBM SPSS Statistics, USA). The means and standard deviation of the samples analyzed were computed while comparison was performed using one‐way analysis of variance (ANOVA). Conversely, the statistical significant difference of all the samples analyzed were performed at *α* = 0.05.

## 3 RESULTS AND DISCUSSION

### 3.1 The chemical composition of the native and modified quality protein maize starches

The starch yields of the modified QPM starch are presented in Table 1. The yield of the native starch (43.80% (wb) was lower than the values (45.70‐60.80%) reported by Ashogbon and Akintayo (2012) for several rice cultivars grown in Nigeria. Paraginski et al. (2014) on the other hand reported starch yields of 45.99 to 66.94% for freshly harvested and stored maize starches, while, Lawal (2004) declared starch yield of 30 to 62.32% for new cocoyam starch. The variation of starch yield may be attributed to the origin and genetic difference of starch source, maturity stage of crop, and extraction method employed in isolating the starches.

**Table 1:** The yield of the modified quality protein maize starch

Starches	%Yield
ACM	94.39^b^ ± 0.46
ATM	88.22^d^ ± 0.93
OXM	90.06^c^ ± 0.80
PGS	98.34^a^ ± 0.47

All the values are means and standard deviation of triplicate determination. Means within the same column having the same superscript are not significantly different at (*P* > 0.05).

ACM, acetylated quality protein maize starch; ATM, acid‐thinned quality protein maize starch; NTS, native quality protein maize starch. OXM, oxidized quality protein maize starch; PGS, pregelatinized quality protein maize starch.

For the modified starches, the yields were 98.34% for pregelatinized sample, while acetylated, oxidized, and acid thinned starches were 94.39, 90.06, and 88.22% respectively. Awolu and Olofinlae (2016) reported yield of 85.30, 97.30, and 90.83% for acid thinned, acetylated, and oxidized water yam starches, respectively. Lawal (2004) also reported a lower yield in oxidized barley starch which resulted into a loss of mass, as it was also suspected to have occurred during the modification of the native starch.

The chemical composition and amylose content of native and modified quality protein maize starch are presented in Table 2. The moisture content of the native and modified QPM starches ranged from 4.56 to 9.20% which was observed for acetylated and native QPM starches, respectively, although, the moisture contents were low and acceptable. Awolu and Olofinlae (2016) reported moisture content values between 6.4 and 11.65% for native and modified water yam starches, Olatunde et al. (2017) revealed the moisture content of banana and plantain modified starches to be within the range of 9.10 to 15.40%. Moisture content of starches depends largely on the method and extent of drying and also the humidity of the surrounding atmosphere (Lawal, 2004).

**Table 2:** Chemical composition of the native and modified quality protein maize starch

Starches	%Moisture	%Ash	%Fat	%Protein	%Amylose
ACM	4.56^e^ ± 0.90	0.46^c^ ± 0.04	0.26^d^ ± 0.84	1.22^c^ ± 0.04	20.03^c^ ± 0.51
ATM	8.40^b^ ± 0.41	0.40^d^ ± 0.43	0.32^c^ ± 0.64	1.13^d^ ± 0.53	21.35^b^ ± 0.67
OXM	5.09^d^ ± 0.42	0.51^b^ ± 0.11	0.43^b^ ± 0.53	1.38^b^ ± 0.18	19.92^d^ ± 0.45
PGS	6.00^c^ ± 0.71	0.67^a^ ± 0.10	0.25^d^ ± 0.80	1.25^c^ ± 0.62	24.24^a^ ± 0.98
NTS	9.20^a^ ± 0.86	0.65^a^ ± 0.13	0.55^a^ ± 0.23	1.63^a^ ± 0.03	24.41^a^ ± 0.72

All the values are means and standard deviation of triplicate determination. Means within the same column having the same superscript are not significantly different at (*P* > 0.05).

ACM, acetylated quality protein maize starch; ATM, acid‐thinned quality protein maize starch; NTS, native quality protein maize starch; OXM, oxidized quality protein maize starch; PGS, pregelatinized quality protein maize starch.

The moisture content value of 6.95 to 13.24% for native and modified potato starches was reported by Nadir et al. (2015) while a moisture level of 9.82 and 10.20% for arrowroot starch and cassava starch, respectively, was also reported (Raja & Sindhu, 2000). Ashogbon and Akintayo (2012) obtained a moisture value between 10.40 and 12.77% for rice starches. The modified QPM starches had significantly lower moisture content which can impact an increased storage life compared to the native starch since high moisture is an index of deterioration. The native QPM starch had an ash content of 0.65% and it was noticed that there was a decrease in the ash content of the starch after chemical modification. This is an implication that the native starch minerals were degraded. There was no significant difference (*P* > 0.05) in the ash contents of the native and pregelatinized QPM starch.

Several authors have studied starches from different botanical sources and all obtained varying levels of ash contents (Ashogbon & Akintayo, 2012; Awolu & Olofinlae, 2016; Nadir et al., 2015; Olatunde et al., 2017). The protein content of the native and modified QPM starches is ranged between 1.13 and 1.63%, the values are a little on the high side as significant level of protein found in starches is a function of its purity.

The high level of protein observed for the native QPM starch is as a result of the starch isolation method used for this study and also botanical source of the QPM, Cisse et al. (2013) reported the protein level of yellow and white QPM grown in Côte d'Ivoire were between 0.35 and 0.37%. Also, Awolu and Olofinlae (2016) reported a protein level of below 1% for water yam starch. Olatunde et al. (2017) had a high level of protein of between 2.10 and 2.44% for banana and plantain starches. Nadir et al. (2015) also recorded a protein values between 0.17 and 0.40% for potato starch, while Ashogbon and Akintayo (2012) reported from 0.40 to 0.43%.

Lipid content of the native QPM starch was 0.55%. However, the modification process reduced the lipid content of the QPM starch. This reduction in the lipid content of the starches makes it a useful ingredient in the formulation of a low‐fat food products. The amylose content of the native and modified QPM ranged from 19.92 to 24.41%, with the lowest and highest values observed for oxidized and native QPM starches, respectively. It was also detected that the chemical starch modifications procedure used for this study significantly (*P* ≤ 0.05) reduced the level of amylose content of acetylated, acid‐thinned, and oxidized starches. Cisse et al. (2013) reported an amylose content ranging from 24.82 to 25.11% for native and modified two varieties of QPM in Cote‐d'lvoire, while Awolu and Olofinlae (2016) and Nadir et al. (2015) also reported reduction in the amylose contents of modified starches of water yam and potato starches, respectively.

### 3.2 Acetyl content, degree of substitution of acetylated starch, carbonyl and carboxyl content of oxidized quality protein maize starches

The acetyl content and degree of substitution (DS) of acetylated quality protein starch were 0.022 and 0.07, respectively. It was reported by Lawal and Adebowale (2005) that the occurrence of hydrogen bonds present in acetylated starch is restricted due to electrostatic repulsion forces in the starch molecule, furthermore, acetylated starches which possess a low DS (0.01 to 0.2) has numerous functions it can perform in the industries. Some examples include using acetylated starch as adherents, thickeners, stabilizers, texturizers, and encapsulation agents (Elomaa et al., 2004). The moles of acetyl substituent per mole of d‐glucopyranose unit expresses the DS of acetylated starches (Elomaa et al., 2004).

The DS should be less than 0.025 to be applicable for industrial use (Awolu & Olofinlae, 2016). Moreso, the authors reported an extent of acetylation and acetyl group content of 0.06 and 1.51%, respectively for acetylated water yam starch. Ayucitra (2012) applied various levels of acetylation to maize starch and obtained varying values for acetyl group and extent of acetylation in the range of (2.16 to 5.29%) and (0.08 to 0.21), respectively while, Mirmoghtadaei, Kadivar and Shahedi (2009) obtained values of DS in acetylated starches with acetic anhydride at concentrations of 6 and 8% for oat starch and found the DS to be 0.05 and 0.11, respectively. The extent of acetylation for various starches is primarily influenced by the introduction of acetyl groups into the starch structure, and is simply expressed as DS (Elomaa et al., 2004).

The carbonyl and carboxyl contents of oxidized QPM starch is 0.060 and 0.039, respectively. It was observed that the carbonyl content was higher than the carboxyl content for the oxidized QPM starch. The increase in the carbonyl group is attributed to the high pH of the hypochlorite used for oxidation procedure (Awolu & Olofinlae, 2016). However, the content of the carbonyl group is within the range recommended for safe industrial use by the United States Food and Drug Law Agency. The occurrence of carbonyl and carboxyl groups in oxidized starches was owed to oxidation of the hydroxyl groups of starch molecules to carbonyl groups and then the carboxyl groups and numerous researchers have studied the oxidation of corn, banana, potato, rice, and bean starches with different concentrations of sodium hypochlorite and reported that there was a gradual increase in the carbonyl and carboxyl contents with the increasing concentration of active chlorine (0, 1, and 2 g/100 g) (Fonseca et al., 2015). Awolu and Olofinlae (2016) reported the carboxyl and carbonyl content of oxidized water yam starch to be 0.036 and 0.019, respectively while Fonseca et al. (2015) reported theirs for oxidized potato starch to be within the range of 0.068 to 0.139 and 0.039 to 0.067 for several concentrations of hypochlorite.

### 3.3 Functional properties of quality protein maize starch and derivatives

The functional properties of the native and modified QPM starch are presented in Table 3. The bulk density of the samples ranged from 0.59 to 0.88 g/cm^3^ as it was observed that pregelatinized starch had the highest bulk density value of 0.88 g/cm^3^ while acetylated starch had the least value of 0.59 g/cm^3^. The Bulk density is expressed as an index of packaging as well the degree of coarseness of a starch or flour sample. Pregelatinized starch was the coarsest of the starch samples, in contrast, the acetylated QPM starch sample was very smooth. Significant difference (*P* ˂ 0.05) was observed among the starch samples in terms of their bulk density. The coarseness of the starch samples was reduced during chemical modification while the physical method of modification employed for this study enhanced it, as reported by Awolu and Olofinlae (2016) for modified water yam starches.

**Table 3:** Functional properties of native and modified quality protein maize starches

Starch	Bulk density (g/cm^3^)	WAC (g/g)	OAC (g/g)	viscosity (cp)	pH	Reducing Sugar (mg/100mL)	Dispersibility (%)	Sedimentation (%)
ACM	0.59^e^ ± 0.19	3.35^a^ ± 1.17	1.84^a^ ± 0.70	2.50^c^ ± 1.14	6.45^b^ ± 1.57	260.50^d^ ± 1.70	72.50^c^ ± 1.94	15.50^c^ ± 1.70
ATM	0.64^d^ ± 0.11	2.20^b^ ± 1.14	1.66^c^ ± 0.17	2.00^e^ ± 1.07	4.50^e^ ± 1.50	288.50^b^ ± 1.10	74.50^b^ ± 2.98	16.50^b^ ± 1.85
OXM	0.68^c^ ± 0.23	1.55^d^ ± 1.07	1.75^b^ ± 0.11	2.15^d^ ± 1.03	6.75^a^ ± 1.91	273.50^c^ ± 2.70	78.50^a^ ± 2.71	14.50^d^± 1.10
PGS	0.88^a^ ± 0.10	1.95^c^ ± 1.71	1.37^e^ ± 0.34	2.99^a^ ± 1.14	5.45^c^ ± 1.65	288.00^b^ ± 1.81	72.50^c^ ± 2.70	17.26^a^ ± 0.74
NTS	0.72^b^ ± 0.90	1.85^c^ ± 1.07	1.45^d^ ± 0.85	2.75^b^ ± 1.21	5.15^d^ ± 1.00	308.00^a^ ± 1.41	75.50^a^ ± 2.56	17.50^a^ ± 0.70

All the values are means and standard deviation of triplicate determination. Means within the same column having the same superscript are not significantly different at (*P* > 0.05).

ACM, acetylated quality protein maize starch; ATM, acid‐thinned quality protein maize starch; NTS, native quality protein maize starch; OXM, oxidized quality protein maize starch; PGS, pregelatinized quality protein maize starch.

The water absorption capacity and oil absorption capacity of the native and the modified quality protein starches were 1.55 to 3.35 g/g and 1.37 to 1.84 g/g, respectively. It was observed that acetylation, acid thinning, and pregelatinization increased the water absorption capacity of the native starch owing predominantly to the integration or alteration of certain functional groups present in the native starch molecule leading to an increase in the binding capacity. However, oxidized starch showed a reduction in water absorption capacity. Modification also improved the oil absorption capacity of the native starch except in pregelatinized starch where a decrease was observed (1.37 g/g).

The viscosity of the starch samples ranged from 2.00 to 2.75 cP. Chemical modification reduced the viscosity of the starch samples except in pregelatinized starch which had an increase (2.99 cP). The reduction in viscosity may be due to the method of modification adopted for the process. Modified starches are used widely as thickeners and stabilizers in products such as gravies, sauces, soups, sausage fillings, milk‐based foods, and puddings.

The pH of any starch is a significant asset when it comes to industrial applications, because pH is being used generally to indicate the acidic or alkaline properties of liquid media (Ashogbon & Akintayo, 2012). The pH of the native and modified QPM was between 4.50 and 6.75. The pH of the native QPM starch was 5.15, which was slightly acidic and will not pose any problem when used as food additive in an acidic medium. Modifications due to acid treatments reduced the pH, while modifications due to alkaline treatment increased the pH. The reducing sugar ranged from 260 to 308 mg/100 mL with the least value reported in the acetylated QPM starch while the highest value was observed in the native QPM starch. It further implied that modification decreased the levels of reducing sugar in the starch samples. Reducing sugar is responsible for a sweeter taste and texture of the final products.

The dispersibility of the native and modified QPM starches ranged from 72.50 to 78.50%. The highest value was obtained in the oxidized QPM starch whereas the lowest value reported in the acetylated and pregelatinized QPM starches. Modification reduced dispersibilities of QPM starch in this study except in oxidized sample. The implication is that oxidized starch will reconstitute better. Dispersibility is a measure of reconstitution of flour or flour blends in water; the higher the dispersibility the better the flour reconstitutes (Akanbi et al., 2009; Ashogbon & Akintayo, 2012). Ashogbon and Akintayo (2012) obtained values between 75.10 and 82.12% for rice starches dispersibilities, whereas, Akanbi et al. (2009) reported 40.67% for breadfruit starch.

The percentage sedimentation volume of native and modified QPM starches was between 14.50 and 17.50%. Pregelatinized QPM starch had the highest sedimentation value (17.50%), closely followed by native starch. According to Rafiq et al. (2015), modification reduced sedimentation volume of Horse chestnut starch, which was similar to the trend reported for acetylated, acid‐thinned, and oxidized modified quality protein maize starches in this study. The reduction of sedimentation levels of the modified starches was probably due to the disruption of the granules resulting in low volume make up. In addition, decreased sediment volume may also be due to large starch granules which caused diminution in bond strength upon heating (Rafiq et al., 2015).

### 3.4 Effect of temperature on the swelling power and percentage solubility on the native and modified quality protein maize starches

**Figure 1:**
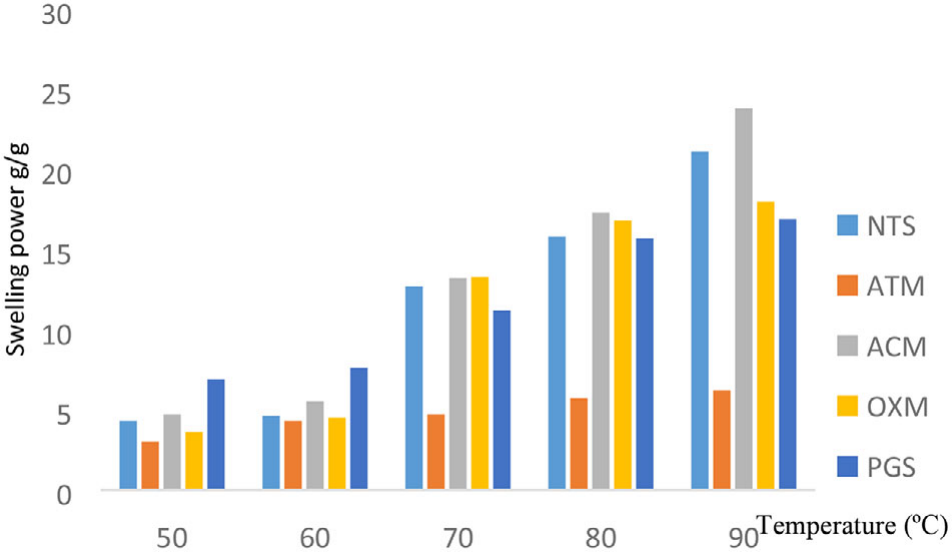
Effect of temperature on swelling power of native and modified quality protein maize starch.

The swelling power for the native and modified starches at temperatures ranging from 50 to 90 °C is shown in Figure 1. From the study, the swelling power of the starches rose as the temperature increased. This phenomenon was expected, which is an indication that absorption of water by a starch granule can be elevated by increasing temperature. Acetylated QPM starch exhibited the highest swelling power of 23.80 g/g at 90 °C while the lowest swelling power was noted for acid thinned QPM starch (3.00 g/g at 50 °C). The native QPM starch had a swelling power of 21.10 g/g which was in agreement with the values reported by Sandhu and Singh (2007), where their swelling power at 90 °C was between 13.0 and 20.7 g of water per gram of dry starch in nine maize varieties. The internal bond strength present in the starch granules influences swelling power (Paraginski et al., 2014). Oxidation, acid thinning, and pregelatinization modification reduced the swelling power of the native starch but the opposite was observed for acetylated starch where increment in swelling power was recorded. Acid thinned starch had the least value simply because of the disruption of the amorphous region of the starch during the modification of which several authors reported similar circumstances for acid thinned starches (Ali & Hasnain, 2011; Lawal, 2004).

**Figure 2:**
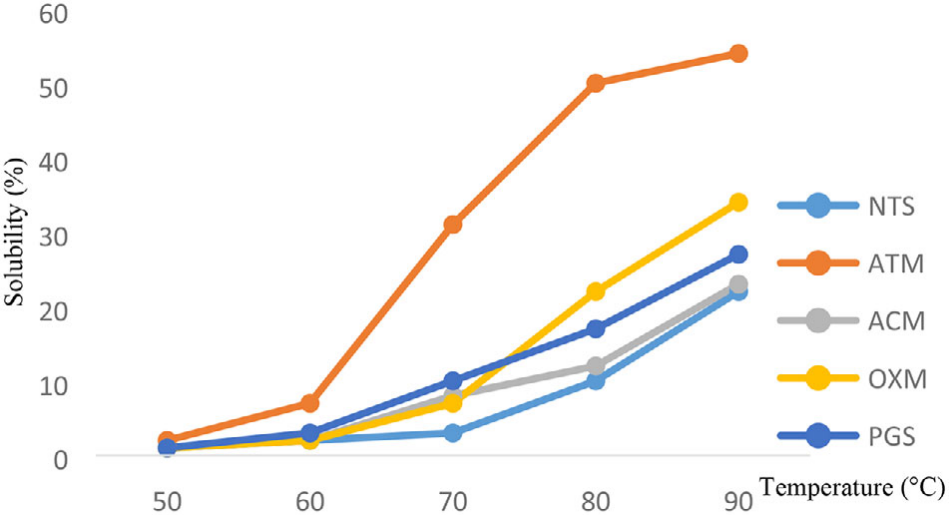
Effect of temperature on percentage solubility of native and modified quality protein starch.

The solubility of native and modified QPM starch is shown in Figure 2 and there was increment in solubility as temperature rose from 50 to 90 °C. Major changes in the solubility were observed for the starches between 70 and 90 °C, as a result of amylose leaching from the starch granule and diffusion during the swelling (Paraginski et al., 2014). Acid thinned starch exhibited the highest solubility at 54% compared to the other starch samples used for this study, in agreement with water yam starch modification reported by Awolu and Olofinlae (2016). Conversely, Adebowale, Olu‐Owolabi, Olawumi, and Lawal (2005) also observed an increase in the solubility of heat moisture treatment of finger millet starch. The elevated solubility and swelling capacities of the starches is attributed to the presence of hydrophilic substituting groups which allows water retention due to their hydrogen bonds forming capacity and this singular factor ensures high retention of water that goes into the granule. Elevated swelling capacity can be a valuable asset in the manufacture of some starch base products.

### 3.5 Effect of pH on the swelling power and percentage solubility of the native and modified quality protein maize starches

**Figure 3:**
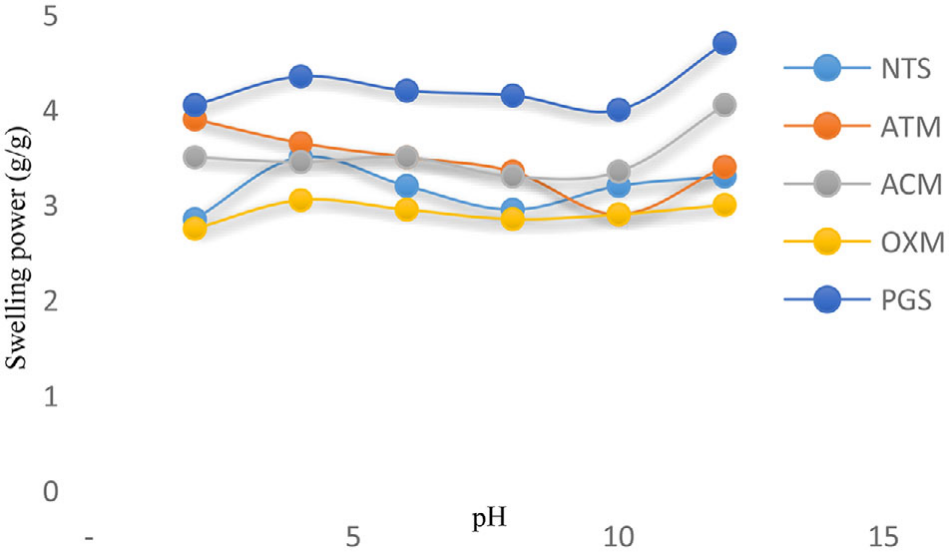
Effect of pH on swelling power of native and modified quality protein maize starch.

**Figure 4:**
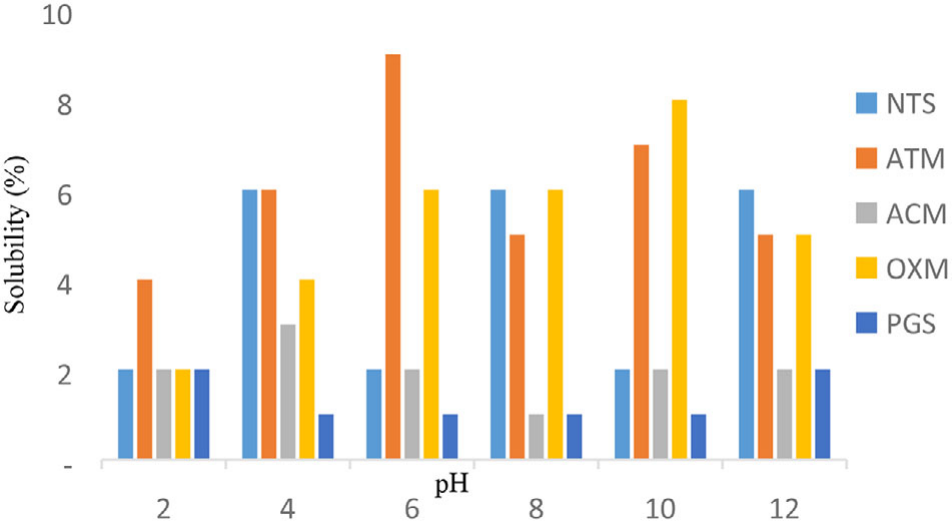
Effect of pH on percentage solubility of native and modified quality protein maize starch.^*^ACM, acetylated quality protein maize starch; ATM, acid‐thinned quality protein maize starch; OXM, oxidized quality protein maize starch; PGS, pregelatinized quality protein maize starch; NTS, native quality protein maize starch.

Charts showing the effect of pH (2 to 12) on swelling capacity and solubility of native and modified QPM are shown in Figures 3 and 4, respectively. The swelling power and solubility index were pH dependent and did not follow any particular trend. The swelling power of the QPM starches increased except for oxidized starch that behaved otherwise. The maximum swelling power for each of the starch samples was evident at pH 12, 12, 2, 4, and 4 for pregelatinized, acetylated, acid‐thinned, native, and oxidized QPM starches, respectively. The highest value for swelling power was observed at pH 12 for pregelatinized QPM starch (4.65) and the lowest was at pH 2 for oxidized QPM starch (2.70). Under alkaline circumstances, the starch may experience partial gelatinization, which result to high solubility at pH 10.0 (Olayide, 2004).

### 3.6 Least gelation concentration of native and modified quality protein maize starches

The results for least gelation concentration for the native and modified QPM starch are presented in Table 4. The occurrence of carbonyl and carboxyl groups as a result of oxidation probably caused intermolecular repulsions which inhibited the interaction of the oxidized starch molecules, and therefore, resulted in decreasing gelation properties (Lawal, 2004). Acid thinning methodology also reduced gelation, as the erosion of the amorphous region by acid hydrolysis resulted in reduced interference of the double helical chains advancing toward each other, so the gelling properties was reduced and more concentration will be needed for proper gelation (Lawal, 2004).

**Table 4:** Least gelation concentration of native and modified quality protein maize starches

Concentration (%)	ACM	ATM	OXM	PGS	NTS
2	‐ No gel	‐ No gel	‐ No gel	‐ No gel	‐ No gel
4	‐ No gel	‐ No gel	‐ No gel	‐ No gel	‐ No gel
6	‐ No gel	‐ No gel	‐ No gel	‐ No gel	‐ No gel
8	‐ No gel	‐ No gel	‐ No gel	‐ No gel	‐ No gel
10	‐ No gel	‐ No gel	‐ No gel	‐ No gel	‐ No gel
12	+ Gel	‐ No gel	‐ No gel	‐ No gel	‐ No gel
14	+ Gel	‐ No gel	‐ No gel	‐ No gel	+ Gel
16	+ Gel	+ Gel	+ Gel	‐ No gel	+ Gel
18	+ Gel	+ Gel	+ Gel	+ Gel	+ Gel
20	+ Gel	+ Gel	+ Gel	+ Gel	+ Gel
LGC	14	16	14	18	12

LCG, least gelation concentration.

ACM, acetylated quality protein maize starch; ATM, acid‐thinned quality protein maize starch; NTS, native quality protein maize starch; OXM, oxidized quality protein maize starch; PGS, pregelatinized quality protein maize starch.

### 3.7 Color parameters of native and modified quality protein maize starches

Summary of the color parameters of the native and modified starches are presented in Table 5. The most significant color change observed was noted for the pregelatinized QPM starch, with *L* ^*^ values of 85.52, *a* ^*^ value of 3.07, and *b* ^*^ values of 14.99 were observed. The values for pregelatinized quality protein starch was significantly (*P* ˂ 0.05) different from the native and chemically modified starches. The low *L* ^*^ value could be a result of the high temperature of pregelatinization of the sample.

**Table 5:** Color parameters for native and modified quality protein maize starches

Starches	*L* ^*^	*A*	*B*
ACM	94.90^a^ ± 1.10	0.85^b^ ± 0.04	9.63^c^ ± 0.21
ATM	92.83^a^ ± 3.26	0.94^b^ ± 0.23	9.46^cd^ ± 0.36
OXM	96.29^a^ ± 2.36	0.49^c^ ± 0.07	8.66^d^ ± 0.40
PGS	85.52^b^ ± 1.89	3.07^a^ ± 0.08	14.99^a^ ± 0.35
NTS	95.23^a^ ± 2.52	0.77^b^ ± 0.19	10.72^b^ ± 0.72

All the values are means and standard deviation of triplicate determination. Means within the same column having the same superscript are not significantly different at (P > 0.05).

ACM, acetylated quality protein maize starch; ATM, acid‐thinned quality protein maize starch; NTS, native quality protein maize starch; OXM, oxidized quality protein maize starch; PGS, pregelatinized quality protein maize starch.

Oxidized QPM starch had the highest *L* ^*^ value at 96.29 but it was not significantly (*P* > 0.05) different from the results obtained for acetylated, acid‐thinned, and native QPM starches. The high value of oxidized modified starch could be due to the interactions between the bleaching agent (hypochlorite) and starch granules. Oxidized QPM starch would be better suited and it can then be suggested that the pregelatinized QPM starch would be better for products requiring very clear starch raw material.

### 3.8 Pasting properties of native and modified quality protein maize starches

The pasting properties of the native and modified QPM starches are presented in Table 6. The application of heat in the presence of water influences the behavior of starch and starch‐based products which brings about distinctive pasting profiles. There is always an explicit relationship proportionality between peak viscosity, swelling power, and breakdown viscosity, also higher level of amylopectin is associated with high swelling capacity (Ashogbon & Akintayo, 2014). The phenomenon of pasting after gelatinization of starch comprises swelling of the granules, molecular components exudation from the granules and complete disruption of the starch granules, while the pasting properties of any starch depends largely on granule size distribution, amylose/amylopectin ratio, and mineral content (Aishat, Adebayo, Busie, & Robert, 2007). Pasting temperature signifies the beginning of viscosity. This temperature is higher than the gelatinization temperature, meaning that starches are completely gelatinized before rise in viscosity (Awolu, 2017, 2019). The pasting temperature of the starches ranged from 79.95 to 84.10 °C. The highest pasting temperature was spotted for oxidized starch (Table 5) as all the methods of modification used for this study increased the pasting temperature of the native starch.

**Table 6:** Pasting properties of native and modified quality protein maize starches

Starches	Peak viscosity (BU)	Trough (BU)	Breakdown (BU)	Final viscosity (BU)	Setback (BU)	Peak time (min.)	Pasting temperature (°C)
ACM	1,437	1151	286	2246	1095	5.60	83.95
ATM	24	10	14	34	24	4.67	83.25
OXM	2,047	1734	313	3099	1365	5.87	84.10
PGS	1,803	1411	392	2031	620	5.60	82.25
NTS	1929	1301	628	2387	1086	5.07	79.95

All the values are means and standard deviation of triplicate determination. Means within the same column having the same superscript are not significantly different at (*P *> 0.05).

ACM, acetylated quality protein maize starch; ATM, acid‐thinned quality protein maize starch; NTS, native quality protein maize starch; OXM, oxidized quality protein maize starch; PGS, pregelatinized quality protein maize starch.

This increment may be as a result of the modification tending to increase the region of crystallinity owing to the reorientation of the starch granules as a strong intragranular bond permits the starch to require more heat before structural disintegration which will in turn lead to the formation of paste. Final viscosity is the ability of starch material to form a viscous paste or gel after cooking or cooling. It is a measure of a particular sample's quality (Awolu et al., 2017; Shimelis, Meaza, & Rakshit, 2006). The final viscosities of the native and modified QPM starches were between 34 and 3,099 (BU). Acid‐thinned starch had the least result while oxidized starch was found to be the highest. Setback viscosities is a measure of the recrystallization of gelatinized starch. The values for setback viscosity for the native and modified were found to be between 24 and 1,365 (BU). It was observed that the setback viscosities were reduced in acid‐thinned and pregelatinized starch samples while oxidation and acetylation increased it. The low setback values could be as a result of structural weakening and disintegration of the starch granules, so the larger the setback value, the higher the retrogradation during cooling and the higher susceptibility of the starch to get stale (Awolu & Olofinlae, 2016).

The hold period (trough) is sometimes referred to as shear thinning, holding strength or hot paste viscosity, is a period when the starch is exposed to a period of continuous heating and application of mechanical shear stress (Kiin‐Kabari, Eke‐Ejiofor, & Giami, 2015). Through viscosity was found to be 10 BU for acid thinned and 1,734 BU for oxidized starch which were the lowest and highest values, respectively. Native QPM starch had a through viscosity of 1,301 BU. Meanwhile, oxidation and pregelatinization increased the through viscosity while acid thinning and acetylation had a reduction effect on it. The breakdown viscosity is a measure of the vulnerability or susceptibility of cooked starch to disintegrate (Ashogbon & Akintayo, 2012). Modification brought about reduction in the breakdown value of the starches when compared with the native starch. The decrease breakdown viscosity for the modified starches is an indication that there is disruption of the amorphous region in the starch granules making amylose have low molecular weight and higher rigidity of starch granules (Paraginski et al., 2014).

### 3.9 Fourier transform infrared spectroscopy (FTIR) results for native and modified quality protein maize starches

The functional groups identified from the FTIR of the native and modified QPM are presented in Tables 7 to 11. In identifying functional groups, wavelengths from 3,500 to 1,500 cm^−1^ were used. Regions between 600 and 1,500 cm^−1^ are referred to as the fingerprint (Sacithraa, MadhanMohan, & Vijayachitra, 2013). The FTIR spectrum of the native QPM starch are summarized in Table 7. The major functional groups were alcohol, alkane, and alkene. The presence of aliphatic chains in the starch tends to give it a sensory perception of fattiness when used as partial replacers of fat in certain foods (Fasuan, Gbadamosi, & Akanbi, 2018).

**Table 7:** FTIR spectrum of the native quality protein maize starches

S/N	Frequency (cm^–1^)	Spectrum region (%)	Absorbing Feature	Compound class	Intensity
1.	3,396.00	4.5	O–H stretch	Alcohol	Strong, broad
2.	2,926.30	7.1	C–H stretch	Alkane	weak
3.	1,651.00	14	C = C stretch	Alkene	weak

Modifications via pregelatinization, oxidation, and acid‐thinning introduced alkyne (C ≡ H) into their respective FTIR spectrum. The major functional groups as obtained from FTIR spectrum in pregelatinized QPM starch are presented in Table 8. Pregelatinization introduced an alkyne functional group (C ≡ H stretch) to the native QPM starch. In all, there were four functional groups (alcohol, alkane, alkyne, and alkene) identified in pregelatinized QPM starch. Gelatinized starches have been reported to be valuable in the production of ready‐to‐eat meals. In addition, they have been found to be useful puddings, instant lactic mixtures, and breakfast foods to achieve thickening or water retention without employing heat (Egharevba, 2019).

**Table 8:** FTIR spectrum of the pregelatinized starch

S/N	Frequency (cm^−1^)	Spectrum region (%)	Absorbing feature	Compound class	Intensity
1	3,418.00	15.4	O–H stretch	Alcohol	Strong, broad
2	2,926.39	18.9	C–H stretch	Alkane	weak
3	2,164.00	32.8	C ≡ C stretch	Alkynes	variable
4	1,656.31	26.5	C = C stretch	Alkene	weak

The summary of FTIR spectrum for the oxidized QPM starch are presented in Table 9. Just like the case of pregelatinization, four functional groups were identified which were alcohol, alkane, alkyne, and alkene. Oxidized starches have been reported to have high clarity or transmittance, low viscosity, and low temperature stability, and therefore, used in confectioneries for coating candles and sweets which easily melts (Egharevba, 2019).

**Table 9:** FTIR spectrum of the oxidized starch

S/N	Frequency (cm^−1^)	Spectrum region (%)	Absorbing feature	Compound class	Intensity
1	3,386.00	18.75	O–H stretch	Alcohol	Strong, broad
2	2,927.00	12.50	C–H stretch	Alkane	Weak
3	2,166.00	12.50	C ≡ C stretch	Alkynes	Variable
4	1,654.51	25.00	C = C stretch	Alkene	Weak

The functional groups identified in acid‐thinned sample were hydroxyl, alkane, alkyne, and alkene (Table 10) as also observed in pregelatinized and oxidized samples. Acid‐thinned reduced hot‐paste viscosity, improved gelling or gel strength, thereby enhanced textural properties of food materials (Mason, 2009). They have applications in gums and jellies (Egharevba, 2019).

**Table 10:** FTIR spectrum of the acid‐thinned starch samples

S/N	Frequency (cm^−1^)	Spectrum region (%)	Absorbing feature	Compound class	Intensity
1	3,426.00	20.00	O–H stretch	Alcohol	Strong, broad
2	2,927.67	20.00	C–H stretch	Alkane	Weak
3	2,166.00	13.33	C ≡ C stretch	Alkynes	variable
4	1,655.26	13.33	C = C stretch	Alkene	Weak

The functional groups in the acetylated QPM starch (Table 11) were alcohol, alkane, and alkyne. Unlike pregelatinization, oxidation, and acid‐thinning with an additional functional group (alkyne), acetylation had the same functional groups with native starch. Acetylation improves paste clarity and freeze‐thaw stability of starch (Egharevba, 2019).

**Table 11:** FTIR spectrum of the acetylated starch samples

S/N	Frequency (cm^−1^)	Spectrum region (%)	Absorbing feature	Compound class	Intensity
1	3,411.00^i^	20.00	O–H stretch	Alcohol	Strong, broad
2	2,927.43^i^	20.00	C–H stretch	Alkane	weak
3	1,653.10^i^	13.33	C = C stretch	Alkene	weak

There were changes in wavenumbers of the native and modified samples which caused shifts in bands but did not in the total removal or alteration of the functional groups. The changes only indicated that the bands in the modified starch samples were marginally excited (Fasuan et al., 2018).

## 4 CONCLUSIONS

Modification affected the properties examined in this study, though, individual modification technique performed best in different properties studied. While modifications increased QPM starch yield by over 100%, there were significant (*P* ≤ 0.05) reduction in the amylose contents of the modified starches with the exception of pregelatinized starch. Also, there were significant (*P* ≤ 0.05) reduction in the viscosities of modified starches except in pregelatinized starch. However, acetylated starch had highest water and oil absorption capacities. While oxidized starch had the best peak, trough, and final viscosities, acid‐thinned starch had the best breakdown and setback viscosities. The results of the FTIR indicated that the major functional groups identified in the starches includes alkanes, alkenes, alkynes, and hydroxyl. Modifications, therefore, improve the physicochemical characteristics of the QPM starch.

## AUTHOR CONTRIBUTIONS

Awolu Olugbenga conceived the research, carry out the experimental design, and supervised the research and manuscript preparation; Joshua Wisdom Odoro collected data and drafted the manuscript; Jumoke Bukola Adeloye proof read the manuscripts; while Oluranti Mopelola Lawal tested the data, and corrected the manuscript.

## CONFLICTS OF INTEREST

The authors declare there are no conflicts of interest.
